# A rare case of unruptured extracardiac multiple sinus of Valsalva aneurysms originating from the orifices with partial aortic wall defects

**DOI:** 10.1186/s40792-019-0608-7

**Published:** 2019-03-27

**Authors:** Nobuhisa Ohno, Kentaro Watanabe, Toshi Maeda, Otohime Kato, Go Ueno, Kosuke Yoshizawa, Keiichi Fujiwara

**Affiliations:** 1 Department of Cardiovascular Surgery, Hyogo Prefectural Amagasaki General Medical Center, 2-17-77 Higashinaniwa-cho, Amagasaki, Hyogo 660-8550 Japan; 2 Department of Cardiovascular Surgery, Shizuoka Children’s Hospital, 860 Urushiyama, Aoi-ku, Shizuoka, Shizuoka 420-8660 Japan

**Keywords:** Aneurysm, Aortic annulus, Atherosclerosis, Extracardiac, Sinus of Valsalva

## Abstract

**Background:**

Sinus of Valsalva aneurysm (SVA) is relatively rare and commonly reported as a congenital anomaly. It is usually found in a single Valsalva sinus protruding into another cardiac chamber and is termed as intracardiac SVA. The aneurysm usually originates from the Valsalva sinus itself, and an orifice of the aneurysm is observed surrounded by the aortic wall. Thus, extracardiac multiple SVAs originating from the orifices with partial aortic wall defects are extremely rare. We report a very rare case of unruptured extracardiac SVAs in both left and right coronary sinuses originating from the aortic annulus.

**Case presentation:**

A 76-year-old Japanese male was diagnosed with enlarged Valsalva sinuses by transthoracic echocardiography during follow-up for peripheral artery disease. Five years after careful observation, gradual SVA enlargement and moderate aortic insufficiency were observed. He underwent modified Bentall’s procedure, with an uneventful postoperative course. Intraoperatively, SVAs were found in the left lateral half of the left and right coronary sinuses of Valsalva on both sides of the commissure between the left and right coronary cusps. Aortic walls were missing at the SVA floor adjacent to the aortic annulus. Pathological examination revealed only mild atherosclerotic changes of the aortic wall near the SVAs. The cause was estimated as either focal degeneration of the sinuses of Valsalva just above the aortic annulus or congenital anomaly, or combination of both of them.

**Conclusions:**

We report on the case of unruptured extracardiac multiple SVAs missing aortic orifice just above the annulus. No similar case presentation was found in the literature. In this paper, we present details of operative findings and procedures, which will aid in procedure selection.

## Background

Sinus of Valsalva aneurysms (SVAs) are usually reported as congenital findings extending into other cardiac structures and most commonly involve the right coronary sinus (RCS) [1, 2]. Conversely, extracardiac unruptured SVAs are very rare, and majority of the cases are described as multiple or single SVAs involving mainly the RCS and the noncoronary sinus (NCS) in relatively young patients with connective tissue disorders [3–5]. There are few reports of extracardiac unruptured multiple SVAs in elderly patients with atherosclerosis alone. In this study, we present an extremely rare case of extracardiac unruptured multiple SVAs in the left coronary sinus (LCS) and the RCS originating from the aortic annulus.

## Case presentation

**Fig. 1:**
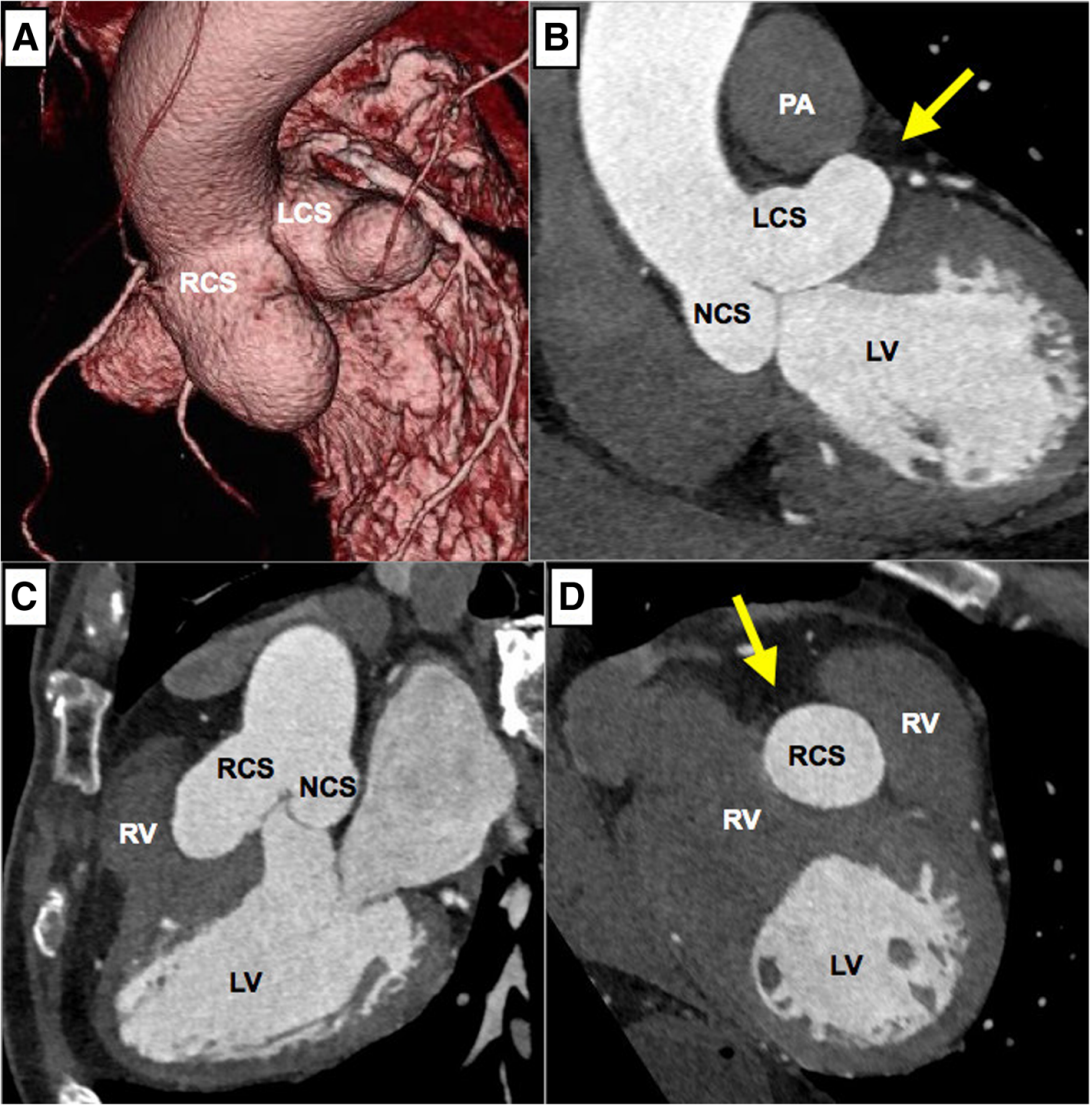
Three-dimensional (**a**) and multiplanar (**b**–**d**) reconstruction by electrocardiogram-gated cardiac computed tomography showing extracardiac aneurysms in the left and the right coronary sinuses. Those aneurysms press aside the pulmonary artery and the right ventricle and bulge out on the surface of the heart (arrows). LCS, left coronary sinus; RCS, right coronary sinus; NCS, noncoronary sinus; LV, left ventricle; RV, right ventricle; PA, pulmonary artery

A 76-year-old Japanese male with a history of hypertension, dyslipidemia, and peripheral vascular disease had previously undergone successful endovascular treatment for occlusive right common iliac artery at 71 years of age. At the time, transthoracic echocardiography performed for screening revealed a dilated aortic root with a width of 49 mm. Subsequent electrocardiogram-gated cardiac computed tomography (EG-CT) revealed SVAs in both the LCS and RCS with no obstruction to surrounding cardiac structures. The sizes of the SVAs from the center of the aorta were 27.2 and 33.1 mm for LCS and RCS, respectively. Due to the relatively small size, they were observed carefully for 5 years, at which time the follow-up EG-CT (Fig. 1) revealed that the LCS and RCS sizes had grown to 34.5 and 35.7 mm, respectively, and transthoracic echocardiography showed moderate aortic regurgitation. The SVA in the LCS was grown between the pulmonary artery and the left atrium and bulged out on the anterior surface of the heart (Fig. 1b). The SVA in the RCS has also grown pressing the right ventricular outflow tract (Fig. 1c). Since both SVAs had been expanding on the surface of the heart (Fig. 1b, d), we concluded that they were extracardiac SVAs. Given that the SVAs, which were extracardiac type with a relatively high risk of rupture, were expanding gradually, the patient provided inform consent to undergo surgery for SVA removal.

**Fig. 2:**
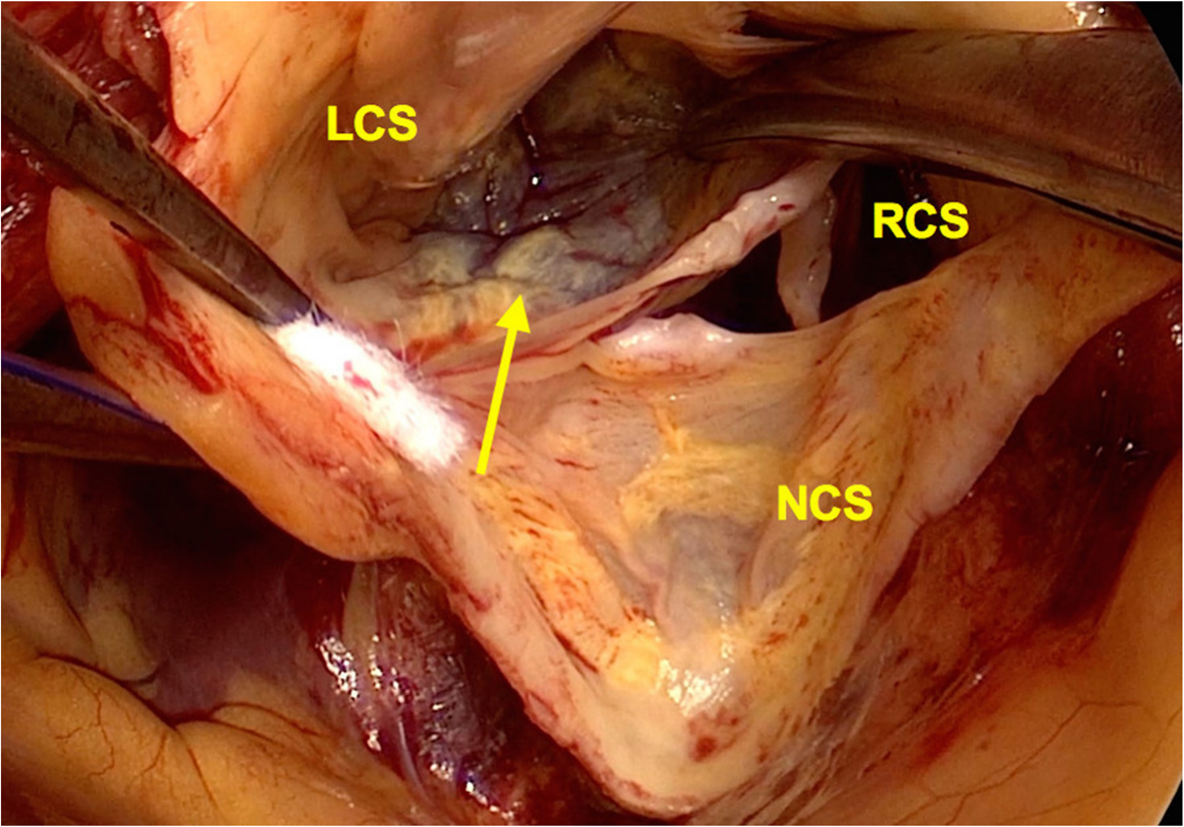
Intraoperative view showing the orifice of the left sinus of Valsalva aneurysm with wall defect of the aortic annulus. Ventricular muscles are observed through a transparent membrane (arrow). LCS, left coronary sinus; RCS, right coronary sinus; NCS, noncoronary sinus

**Fig. 3:**
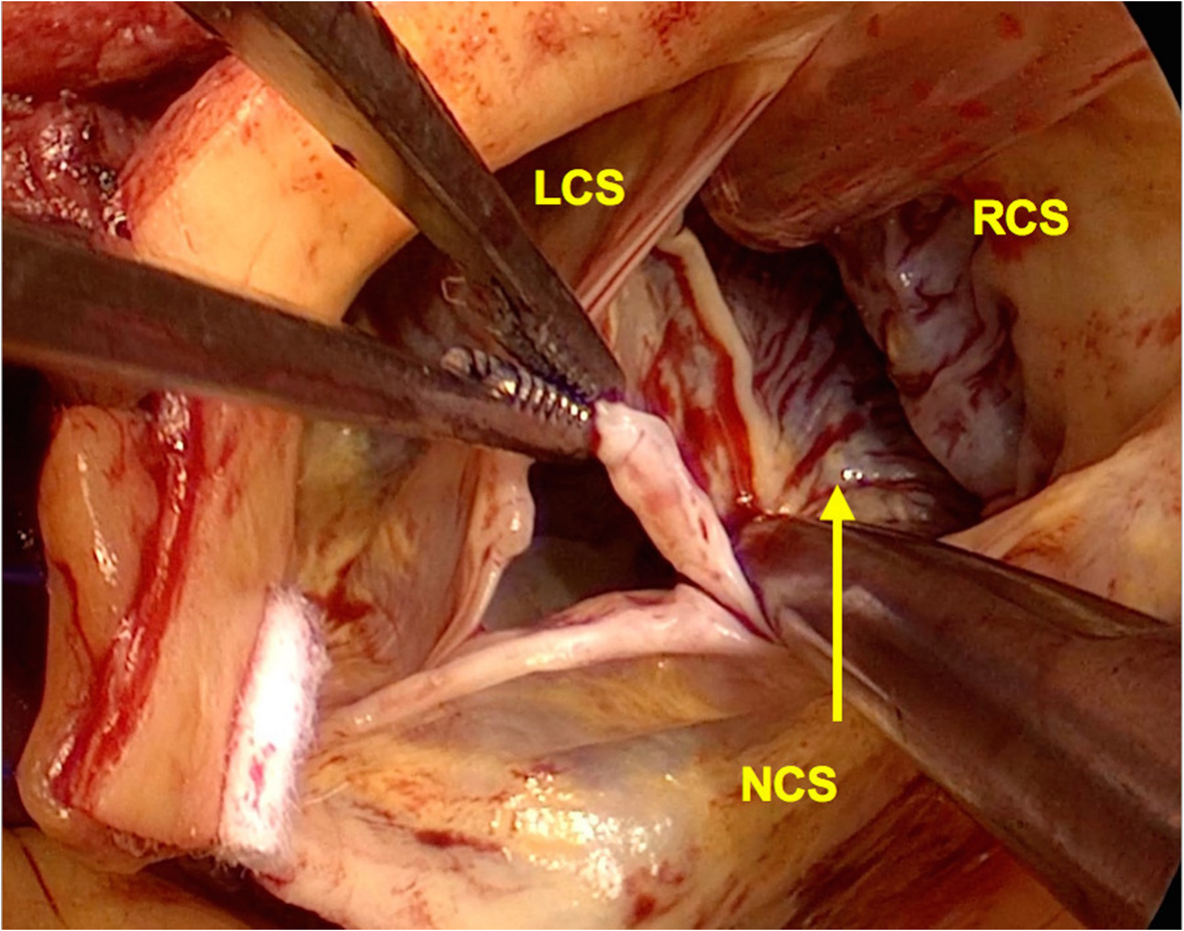
Intraoperative view showing the orifice of the right sinus of Valsalva aneurysm. The aortic wall at the annulus is also lost, and the ventricular muscles are observed (arrow). LCS, left coronary sinus; RCS, right coronary sinus; NCS, noncoronary sinus

The operation was performed via median sternotomy with cardiopulmonary bypass. After cardioplegic arrest, the ascending aorta was transected just above the sinotubular junction. Inspection of the interior of the aortic root revealed SVAs in the left lateral half of the LCS and the RCS on both sides of the commissure between the left and right coronary cusps. Left and right coronary arteries were intact. Aortic walls were missing in the floor of the SVAs adjacent to the aortic annulus, and cardiac muscles could be observed even outside of the annulus through a translucent membrane (Figs. 2 and 3). The SVAs appeared to originate from the orifices with partial aortic wall defects.

The modified Bentall’s approach was performed using a handmade composite graft with a 26-mm polyester graft (Vascutek Gelweave Valsalva graft, Terumo Cardiovascular Systems, Ann Arbor, MI, USA) and a 23-mm bovine pericardial aortic valve (Carpentier-Edwards Perimount Magna Ease, Edwards Lifesciences, Irvine, CA, USA). Pathological examination revealed mild atherosclerotic changes in the aortic wall near the SVAs and inflammatory changes in all aortic valve cusps. The patient recovered well after the surgery and has been well for one and a half years without any events.

## Discussion

SVAs are rare, found in 0.15–1.5% of patients undergoing heart surgery under cardiopulmonary bypass [1]. Majority of the reported SVAs were congenital in origin and are observed during congenital heart surgery. Acquired SVAs are very rare and are caused by infective endocarditis, trauma, systemic inflammatory diseases, connective tissue diseases, and degenerative diseases such as atherosclerosis [3, 6, 7]. Morphologically, SVAs are categorized as intracardiac and extracardiac SVAs. Intracardiac SVAs protrude or rupture into other cardiac compartments, most commonly into the upper portion of right ventricular outflow tract, and can precisely be classified as congenital anomalies [8]. Conversely, extracardiac SVAs, which can arise from a variety of etiologies, do not have a systematic classification. One study summarizing cases of isolated unruptured SVAs [4] indicated that they arose mostly from the RCS and the NCS and that the etiologies included mucoid degeneration (33%), medial necrosis (16%), and atherosclerotic changes (8%). In the current case, histopathological examination showed only mild atherosclerotic changes in the aortic wall around the SVAs, leading us to conclude that these SVAs were the extracardiac type involving multiple sinuses arising from either atherosclerotic changes of the aortic annulus or congenital anomaly or combination of both, which is extremely rare.

There are several reported surgical procedures for SVAs including direct patch closure of the aneurysmal orifice, aortic root reimplantation, and aortic root remodeling [7, 9]. The initial surgical plan included aortic root reimplantation because of the mild damage of the aortic valve. However, the absence of aortic walls in the aortic annulus at the floor of the SVAs ruled out second-row suturing of the reimplantation technique. Additionally, the standard remodeling technique was difficult to perform without an aortic remnant. Conversely, a study on patch closure reported that a single SVA was successfully repaired using sutures with pledgets, which were inserted into the aortic valve annulus from the left ventricular side to the aortic side [10]. Although this technique could possibly be applicable in the current patient, he had two SVAs on both sides of the commissure between the left and right coronary cusps, and preserving the aortic valve geometry after double patch closure was difficult. Considering the age of the patient and the relatively degenerated aortic valve, Bentall’s procedure with an aortic bioprosthesis was used reasonably.

In the current case, several anatomical features of the SVAs were unique. For example, the aortic wall around the aortic annulus, including the commissure between the left and right coronary cusps, was absent. No studies to date described the details of the SVA structure especially those pertaining to the aortic wall attached to the aortic annulus, and the current case illustrates extremely rare anatomical features. Given that the reimplantation or remodeling technique was used for repair in some of the cases, an aortic wall remnant might have been left on the aortic annulus in these patients.

## Conclusion

We herein presented an extremely rare case of unruptured extracardiac multiple SVAs in both the LCS and RCS. The SVAs originating from the aortic annulus were repaired using modified Bentall’s procedure.

## Abbreviations

SVA: Sinus of Valsalva aneurysm
SVAs: Sinus of Valsalva aneurysms
RCS: Right coronary sinus
NCS: Noncoronary sinus
LCS: Left coronary sinus
EG-CT: Electrocardiogram-gated cardiac computed tomography
